# Supplementary material for: Deciphering neuronal variability across states reveals dynamic sensory encoding
Source: Nat Commun. 2025 Feb 19;16:1768. doi: 10.1038/s41467-025-56733-w (PMC11839951; doi:10.1038/s41467-025-56733-w)
Supplement: Supplementary file 1 — Supplementary Information [file 41467_2025_56733_MOESM1_ESM.pdf]

Deciphering neuronal variability across states reveals dynamic sensory encoding

Shailaja Akella*, Peter Ledochowitsch, Joshua H. Siegle, Hannah Belski, Daniel Denman, Michael A. Buice, Severine Durand, Christof Koch, Shawn R. Olsen, Xiaoxuan Jia*

December 26, 2024

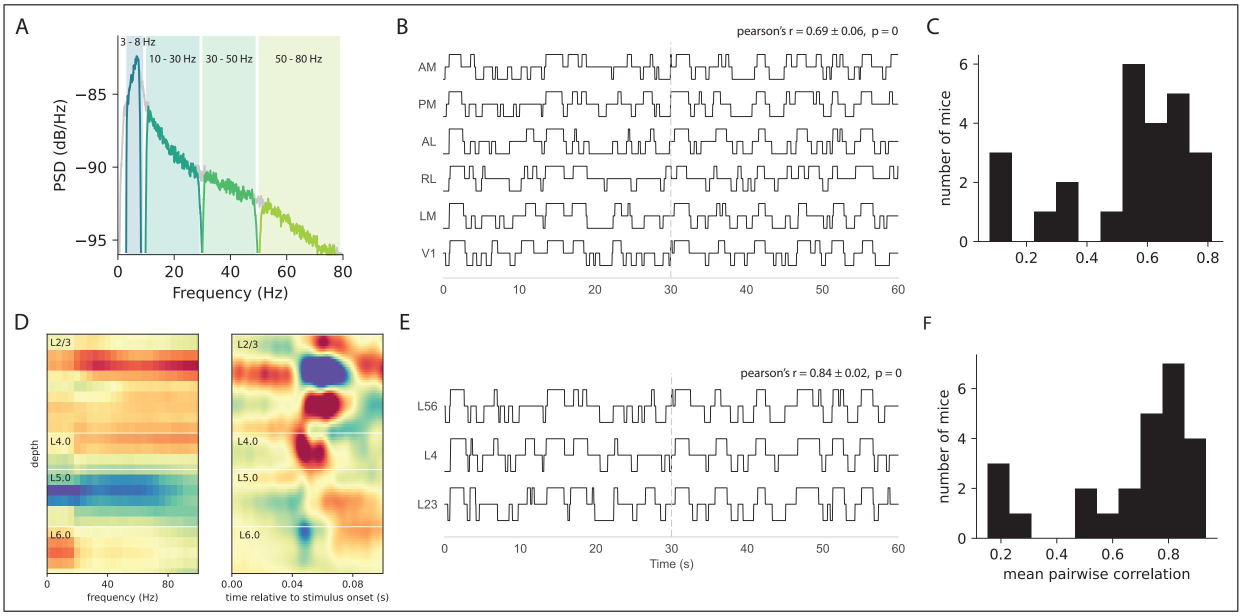

Figure S1: **LFP-based state estimation across brain regions and cortical layers.** **A**, Complete power spectral density (PSD) in a single example channel overlaid with the PSDs of the filtered LFPs in the respective frequency bands. **B**, State sequences estimated using Local field potentials (LFPs) from individual areas of an example mouse. One channel from each layer was incorporated into the Hidden Markov Model (HMM) input matrix. **C**, Histogram summarizing the average pairwise correlations between state sequences estimated from individual areas. **D**, Channel classification into L2/3, L4, or L5/6 based on analyses of the power spectral density (left) and current source density (right) of the LFPs along the cortical depth during the presentation of flashes. **E**, State sequences estimated using LFPs from individual layers of an example mouse. LFPs from all areas were included in the observations provided to the HMM. **F**, Histogram illustrating the average pairwise correlations between state sequences estimated from individual layers.

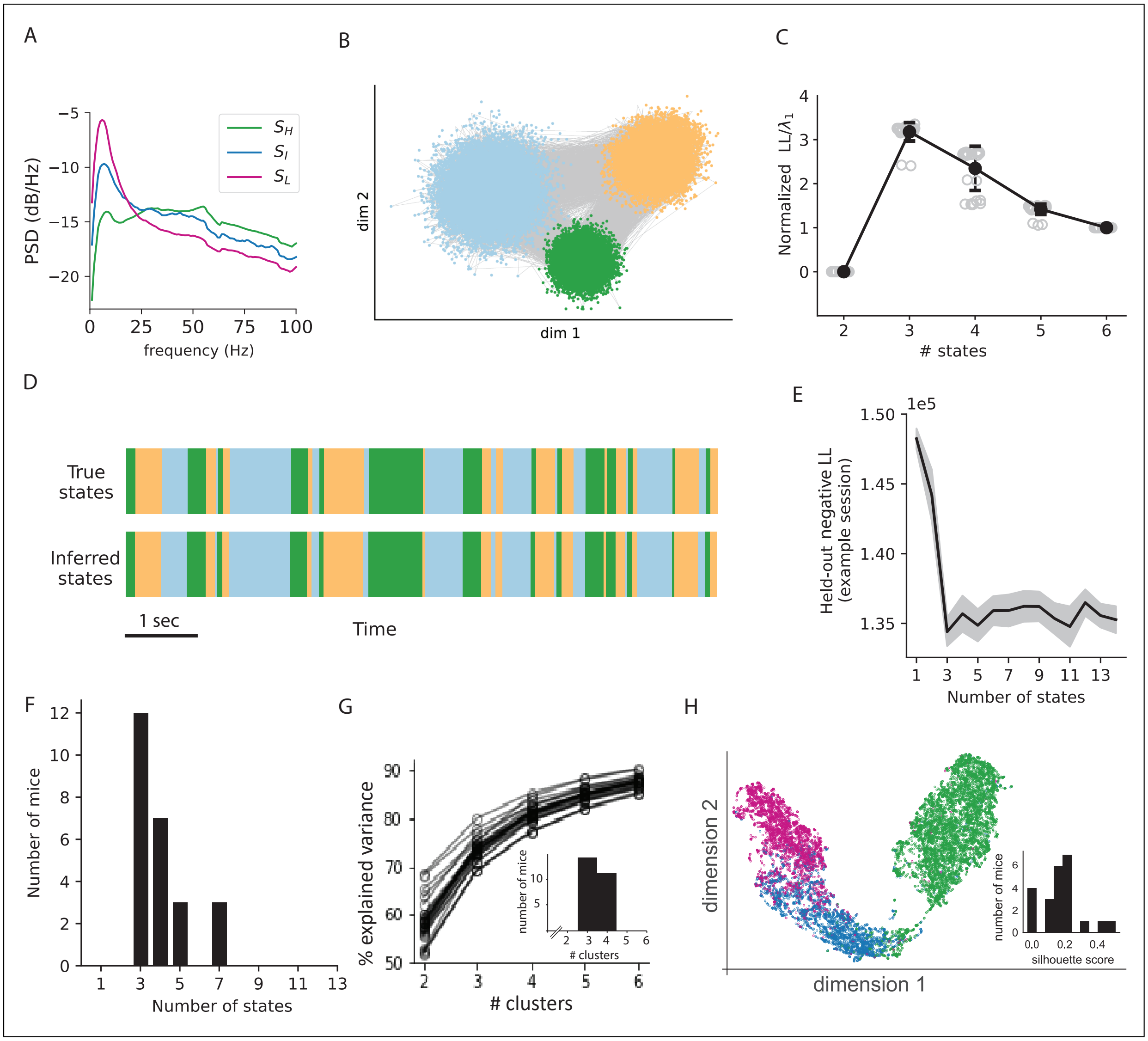

Figure S2: **HMM model selection.** **A**, State-specific power-spectral density of all LFPs in V1 in an example mouse. Such decomposition in all mice further confirmed the spectral distinction observed across the different oscillation states. **B** Simulated three-state HMM time-series, with each color representing a different state. Two dimensions of the 10-dimensional time-series are shown (N = 60,000 samples). **C** Identification of the optimal state from the simulated time-series (n = 30 repeats). **D** Comparison between the inferred states using the HMM and the true states. **E** Model selection for a representative session, showing the minimization of negative log-likelihood. **F** Histogram of the optimal number of states across all mice, determined by identifying the point of maximum curvature in the plot of negative log-likelihood vs. number of states. **G**, K-means clustering to validate the optimal number of states for the Hidden Markov Model (HMM). Elbow method on the variance explained by K clusters. Each line represents data from an individual mouse. (Inset) Histogram of the optimal number of states across all mice **H**, UMAP projection of the LFP provided as observations to the HMM in an example mouse. (Inset) Silhouette scores based on HMM states and UMAP projection.

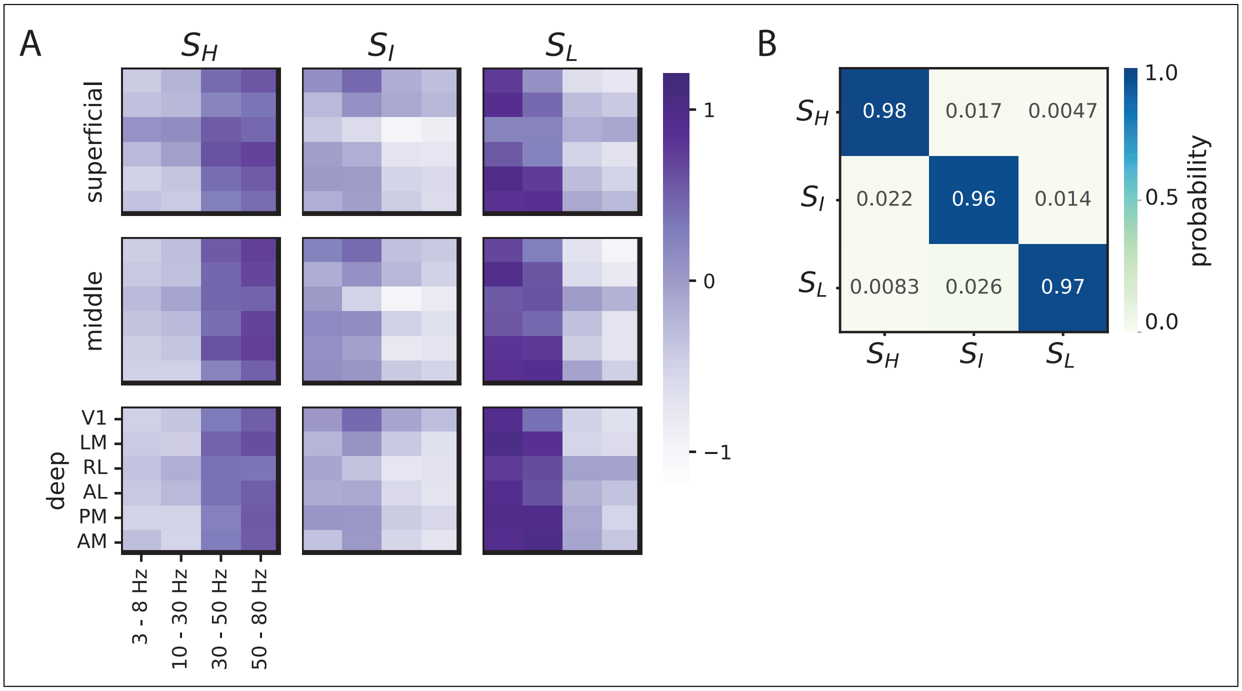

Figure S3: **HMM analysis: State emission and transition probabilities. A**, State emission matrix summarizing the means of all observations within the HMM. **B**, State transition probability matrix. Results from an example mouse.

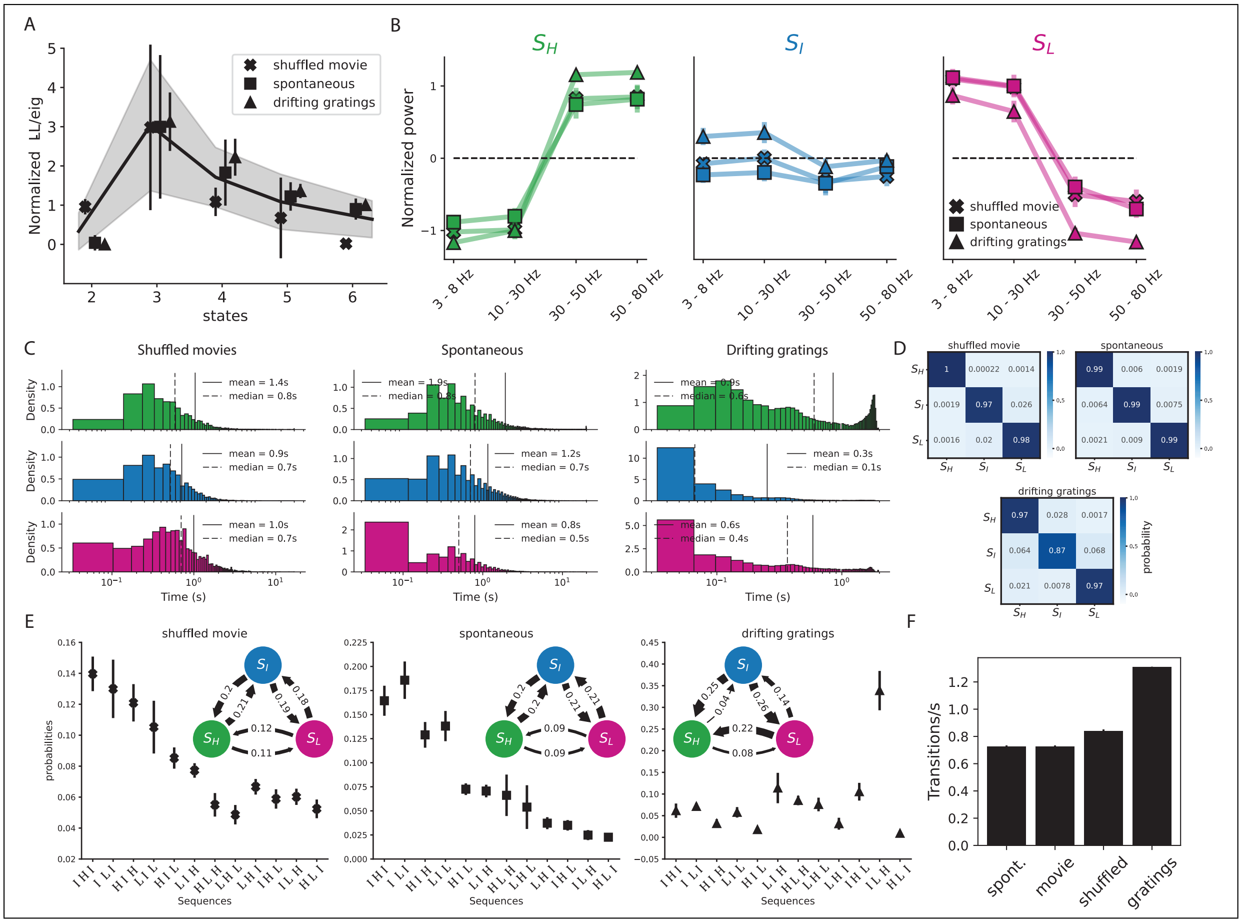

Figure S4: **Oscillation state properties across different stimuli.** **A**, Model comparison among Hidden Markov Models (HMMs) across a range of latent states for different stimulus types (n = 25 mice). Error bars represent s.e.m. **B**, Distribution of LFP power in the three-state model as subjects viewed different stimuli (n = 25 mice). Error bars represent s.e.m.. **C**, Dwell times in each state as subjects viewed various stimuli. **D**, Matrices depicting state transition probabilities. **E**, Average probability of observing 3-step or 2-step (inset) transition sequences to different states while viewing various stimuli. Transition probabilities were calculated from observed sequences averaged across all mice (n = 25 mice). Error bars represent s.e.m. **F**, Number of state transitions per second during the viewing of different stimuli.

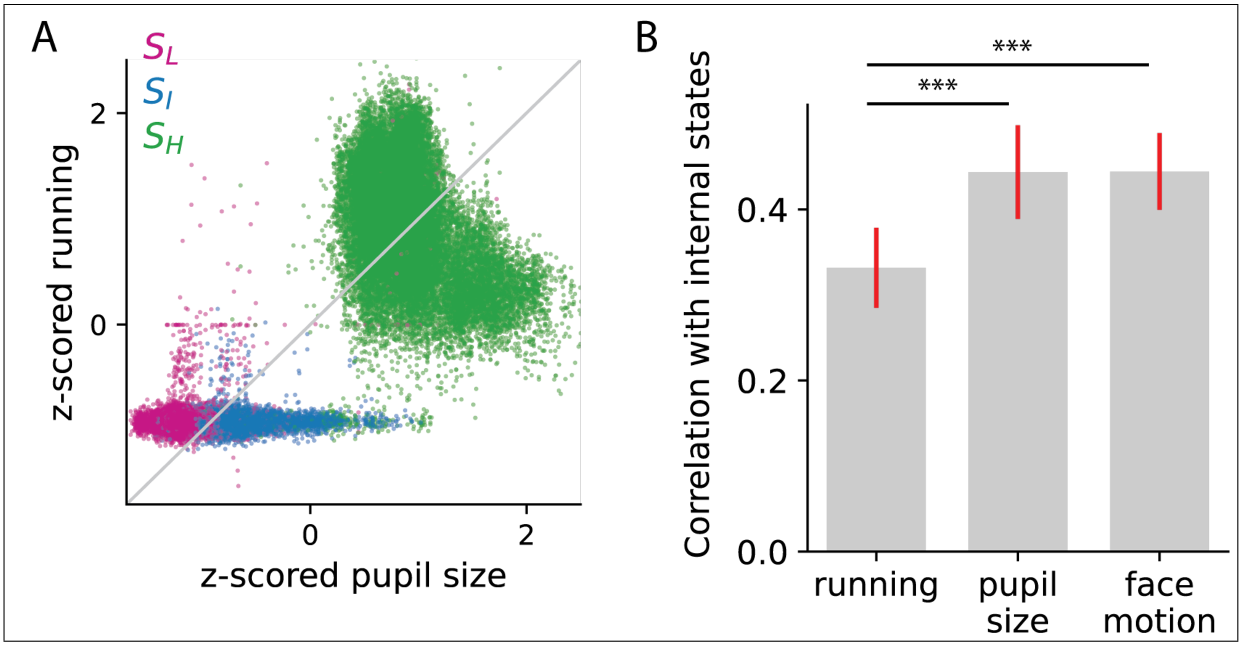

Figure S5: **Relation between oscillation states and behavior.** **A**, Scatter plot of pupil size and running speed color-coded to demarcate the time points of different states. **B**, Average correlation between behavioral states identified individually using running speed, pupil size and facial motion with internal oscillation states (n = 25 mice). Error bars represent s.e.m. Statistical tests were performed using one-way ANOVA and adjusted for multiple comparisons using the Bonferroni correction ($*** : p < 0.0001$, $** : p < 0.001$, $* : p < 0.05$).

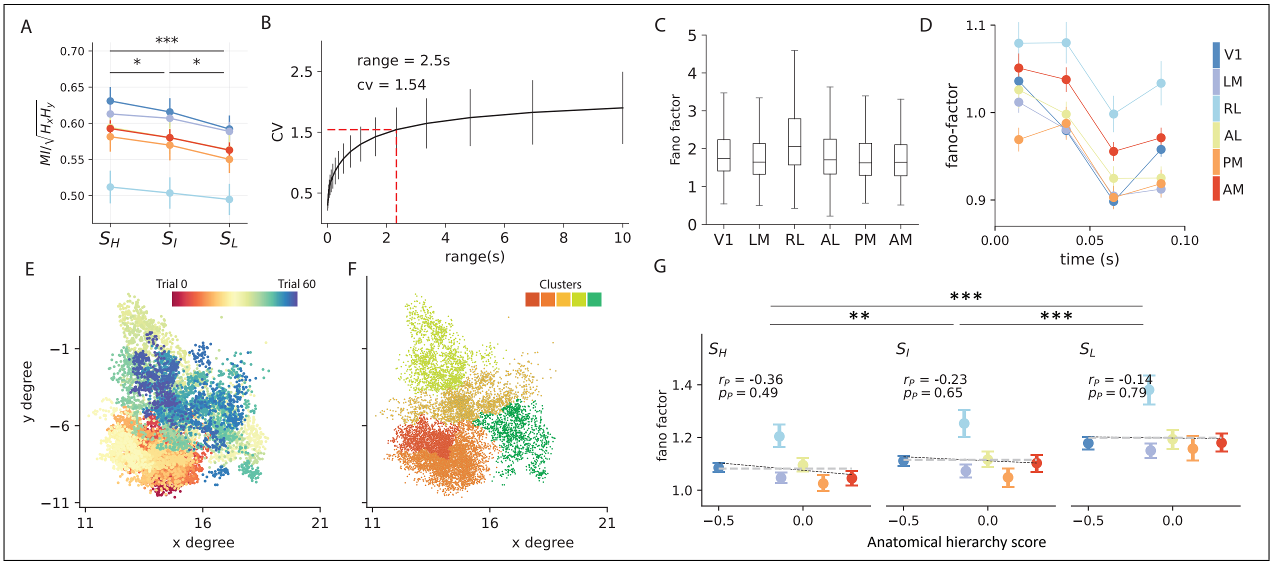

Figure S6: **Control analyses for neuronal variability measures.** **A**, Information encoding along the visual hierarchy across all oscillation states, quantified using mutual information (MI). Error bars represent s.e.m (n = 25 mice). **B**, Time-scale estimation for the construction of inter-spike-interval histograms, utilized in the estimation of the coefficient of variation metric (n = 3923 units). Error bars represent s.e.m. **C**, Box plot summarizing Fano factors in each area (Pearson correlation with anatomical hierarchy scores excluding RL, $r_{p-RL} = -0.7, p_{p-RL} = 0.11$). Box-plot shows the first and third quartiles, the inner line is the median over all neurons (n = 7609 units), and the whiskers represent 1.5 × IQR (Tukey method).**D**, Comparison of Fano factor across visual areas evaluated over time when the mice were exposed to full-field light flashes (n values: V1-1148, LM-502, RL-293, AL-428, PM-256, AM-571 units). **E** Example session showing mouse's gaze on screen colored by trial. **F** Hierarchical clustering applied to mouse's gaze to identify bouts of fixed gaze. **G** FF computed across trials within each cluster of fixed gaze. FF is plotted along the visual hierarchy and across brain states, averaged across all units (One-way ANOVA: p_{S_H,S_I} = 1.1e-03, p_{S_H,S_L} = 5.6e-30, p_{S_I,S_L} = 1.9e-14, n = 5017 units). Pearson correlation with hierarchy scores excluding RL, $S_H : r_{p-RL} = -0.55, p_{p-RL} = 0.33; S_I : r_{p-RL} = -0.28, p_{p-RL} = 0.65; S_L : r_{p-RL} = 0.01, p_{p-RL} = 0.99$. Error bars represent s.e.m. All statistical tests were performed using one-way ANOVA and adjusted for multiple comparisons using the Bonferroni correction ($*** : p < 0.0001$, $** : p < 0.001$, $* : p < 0.05$).

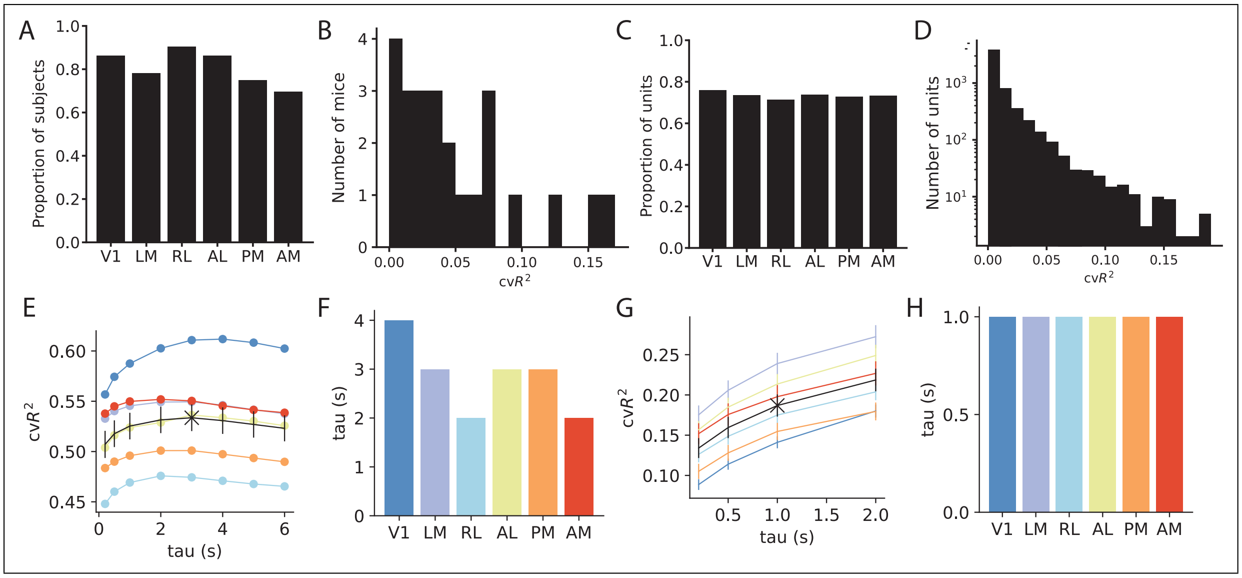

Figure S7: **Impact of oscillation state on spiking variability beyond the influence of other factors.** **A-B** Effect of internal state on averaged population activity. **A** Proportion of subjects showing significant state encoding ($p < 0.05$, ANCOVA). **B** Distribution of cross-validated explained variance (cvR^2) for averaged population activity, with internal states as the predictive feature. **C-D** Effect of internal state on single-neuron activity. **C** Proportion of units in each area significantly influenced by internal state ($p < 0.05$, ANCOVA). **D** Distribution of cross-validated explained variance (cvR^2) for single-neuron activity, using internal states as the predictive feature. **Optimal kernel length selection for regression models.** **E**, Selection of kernel length, τ, for state conditioned regression model to predict variance in the averaged neuronal population activity. The kernel length, which had the maximum predictive power, was chosen. **F**, Optimal kernel length for area-wise state conditioned regression models. **G**, Selection of kernel length, τ, for state-conditioned GLM model to predict single neuron variability. Kernel length was selected on cross validated r^2 using the Elbow method. Results from an example mouse. **H**, Optimal kernel length for area-wise state-conditioned GLM models for the example mouse.

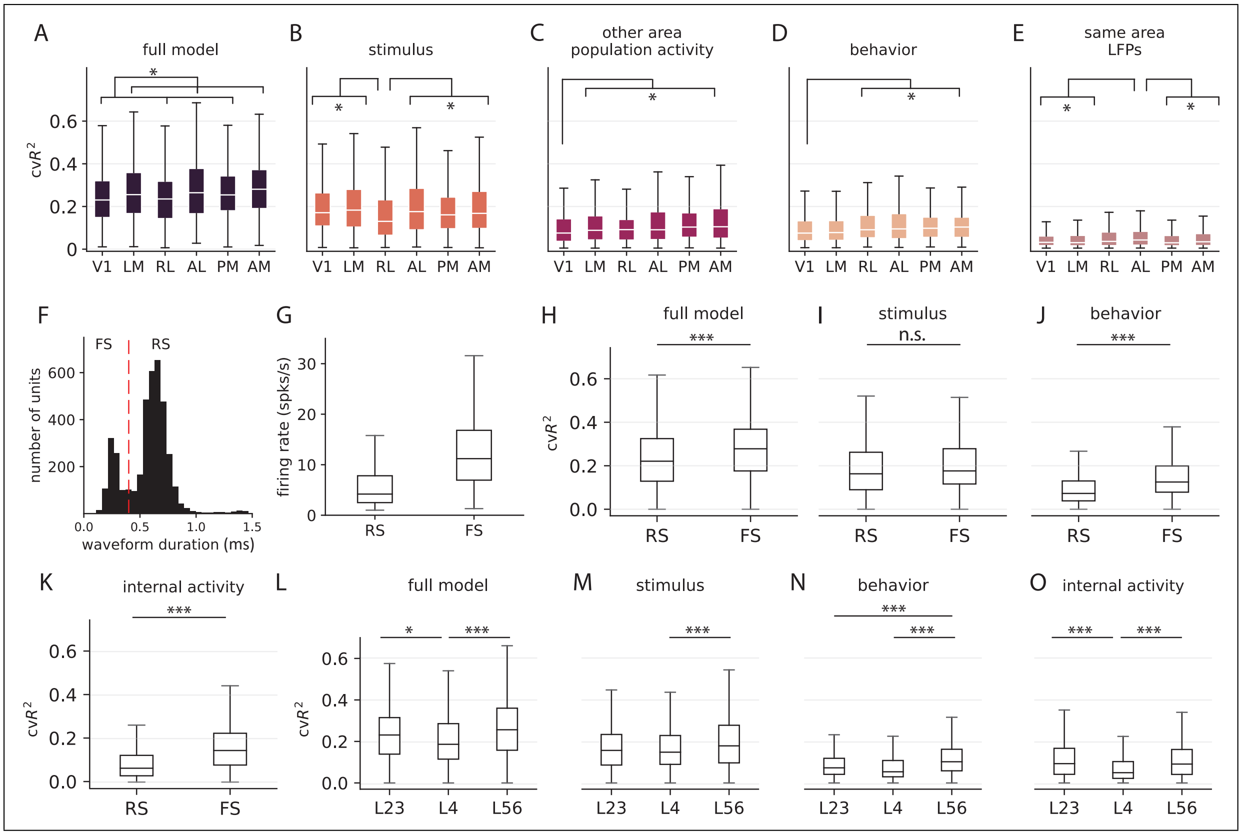

Figure S8: **Relative contributions of the different sources to single neuron variability. A-E**, Contributions from different input features to explain single neuron variance in different visual areas. Analysis of the relative contributions to area-specific single-neuron variability showed that the anterolateral visual areas (LM, AM, and AL) had the highest explained variance of approximately 26.2±0.9% (mean ± std). Consistent with other results, neurons in RL did not encode stimulus features as well as the other visual areas. However, behavior and LFP features explained the most variance in RL and AL neurons (average $\text{cv}R^2_B = 11.7 \pm 0.7\%$, $\text{cv}R^2_{LFP} = 5.0 \pm 0.7\%$, mean ± std), while these features were the least predictive of activity in V1 neurons (average $\text{cv}R^2_B = 9.6\%$, $\text{cv}R^2_{LFP} = 3.6\%$). The predictive power of the averaged neuronal population activity from neighboring areas had trends similar to that observed in the population model, with V1 (average $\text{cv}R^2_P = 9.7\%$) neurons being the least predictive and AM the most predictive (average $\text{cv}R^2_P = 11.8\%$). **F**, Classification of single units into regular spiking (RS) and fast spiking (FS) based on waveform duration. **G-K**, Contributions from different input features to explain single neuron variance across RS and FS cell-types. **L-O**, Contributions from different input features to explain single neuron variance across the cortical depth. Moving across layers, L4 neurons reported the least explained variance, while deep-layer neurons consistently had the highest explained variance across all categories of input features. Box-plots show the first and third quartiles, the inner line is the median over all neurons (n = 3923), and the whiskers represent 1.5 × IQR (Tukey method). Statistical tests were performed using one-way ANOVA and adjusted for multiple comparisons using the Bonferroni correction ($*** : p < 0.0001$, $** : p < 0.001$, $* : p < 0.05$).

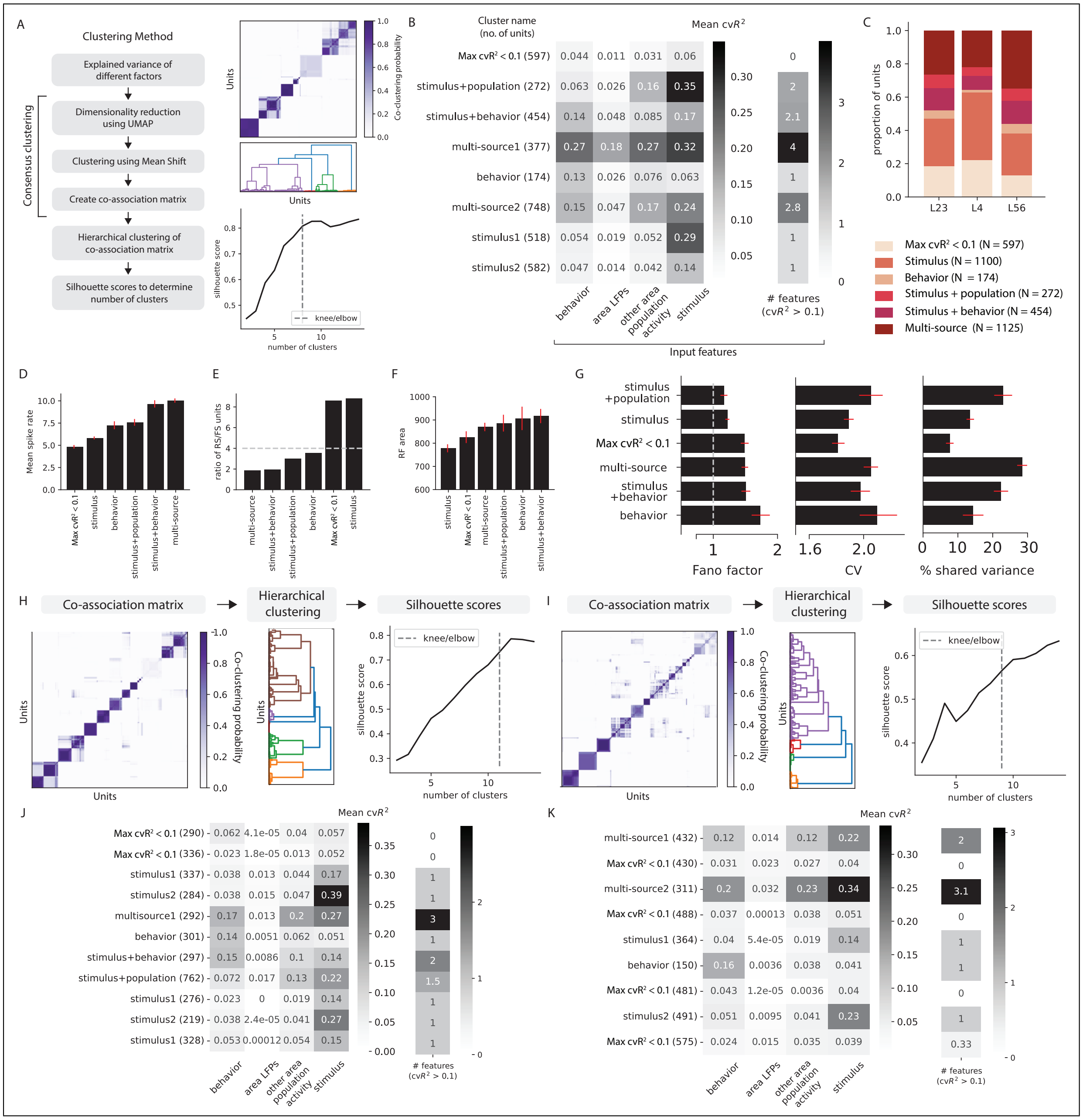

Figure S9: **Heterogeneity of factors influencing visual cortical activity. A** Clustering methods applied to categorize single units into distinct functional clusters based on the dominantly contributing features. **B** Cluster centers corresponding to each identified cluster. Both stimulus-driven clusters (stimulus1 and stimulus2) and multi-source (multi-source1 and multi-source2) clusters were combined for all subsequent analyses. **C** Layer-wise distribution of units within different functional clusters. **D** Mean spike rate of single units across each cluster. **E** Ratio of regular spiking to fast-spiking cells in each cluster. **F** Mean receptive field area of units within each cluster. **G** (left to right) Variability across trials (Fano factor), variability across time (coefficient of variation), and shared variability of neurons within each cluster. Caption continued...

Figure S9: **H-K** Clustering of single units based on state-specific explained variance from individual factors. **H** Left: Co-association matrix constructed using consensus clustering applied to explained variance of units during the high-frequency state. Middle: Hierarchical clustering on co-clustering probabilities. Right: Silhouette scores to determine number of unit clusters in the high-frequency state. **I** Same as **H**, applied to explained variance of units during the low-frequency state. **J** Cluster centers corresponding to identified clusters in the high-frequency state. **K** Same as **J**, in the low-frequency state. Stimulus-driven, multi-source and unexplained clusters were combined for all subsequent analyses. Error bars represent s.e.m.

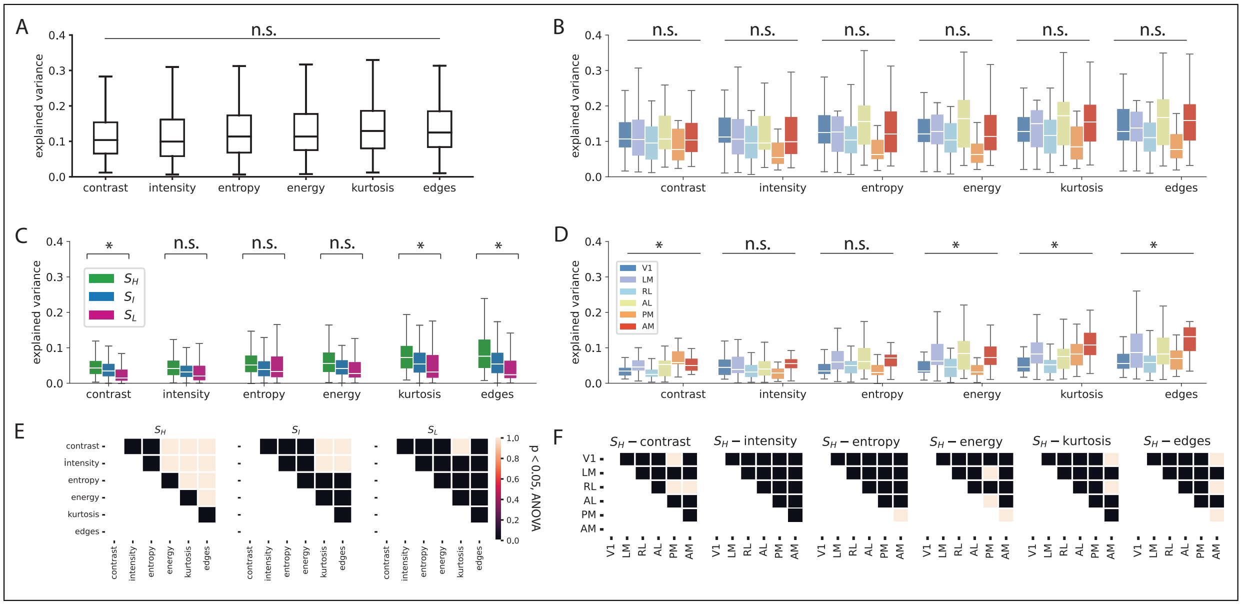

Figure S10: **Relative contributions of the different timulus properties to population-level variability.** **A**, Summary of the variance explained in averaged population activity by different stimulus features. **B**, The contribution of different stimulus features to the variance of averaged population activity across visual areas. **C**, State-wise contributions of different stimulus features to averaged population activity. **D**, Same as **B**, but during the high-frequency state.**E**, Significance results for **C**, one-way ANOVA, p < 0.05, corrected for multiple comparisons. **F**, Significance results for **D**, one-way ANOVA, p < 0.05, corrected for multiple comparisons. Box-plots show the first and third quartiles, the inner line is the median over all mice (n = 25 mice), and the whiskers represent 1.5 × IQR (Tukey method). $*{*}* : p < 0.0001$, $** : p < 0.001$, $* : p < 0.05$.

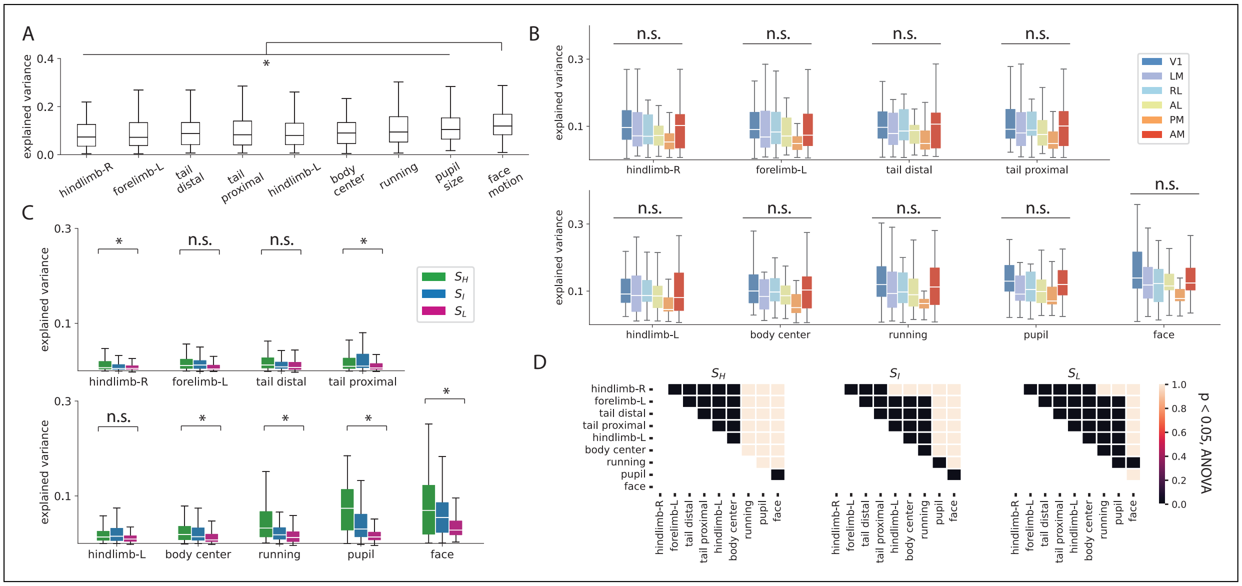

Figure S11: **Relative contributions of different behavior to population-level variability.** **A**, Summary of the variance explained in averaged population activity by various behavioral features. **B**, The contribution of different behavioral features to the variance of averaged population activity across visual areas. **C**, State-wise contributions of behavioral features to averaged population activity. **D**, Significance results for **C**, one-way ANOVA, p < 0.05, corrected for multiple comparisons. Box-plots show the first and third quartiles, the inner line is the median over all mice (n = 25 mice), and the whiskers represent 1.5 × IQR (Tukey method). $*** : p < 0.0001$, $** : p < 0.001$, $* : p < 0.05$.
