# Supplementary material for: Deciphering neuronal variability across states reveals dynamic sensory encoding
Source: Nat Commun. 2025 Feb 19;16:1768. doi: 10.1038/s41467-025-56733-w (PMC11839951; doi:10.1038/s41467-025-56733-w)
Supplement: Supplementary file 2 — Reporting Summary [file 41467_2025_56733_MOESM2_ESM.pdf]

nature portfolio

Corresponding author(s): Shailaja Akella

Last updated by author(s): Dec 27, 2024

Reporting Summary

Nature Portfolio wishes to improve the reproducibility of the work that we publish. This form provides structure for consistency and transparency in reporting. For further information on Nature Portfolio policies, see our Editorial Policies and the Editorial Policy Checklist.

Statistics

For all statistical analyses, confirm that the following items are present in the figure legend, table legend, main text, or Methods section.

n/a	Confirmed	
☐	☒	The exact sample size (*n*) for each experimental group/condition, given as a discrete number and unit of measurement
☐	☒	A statement on whether measurements were taken from distinct samples or whether the same sample was measured repeatedly
☐	☒	The statistical test(s) used AND whether they are one- or two-sided<br>*Only common tests should be described solely by name; describe more complex techniques in the Methods section.*
☐	☒	A description of all covariates tested
☐	☒	A description of any assumptions or corrections, such as tests of normality and adjustment for multiple comparisons
☐	☒	A full description of the statistical parameters including central tendency (e.g. means) or other basic estimates (e.g. regression coefficient) AND variation (e.g. standard deviation) or associated estimates of uncertainty (e.g. confidence intervals)
☐	☒	For null hypothesis testing, the test statistic (e.g. *F*, *t*, *r*) with confidence intervals, effect sizes, degrees of freedom and *P* value noted<br>*Give P values as exact values whenever suitable.*
☐	☒	For Bayesian analysis, information on the choice of priors and Markov chain Monte Carlo settings
☐	☒	For hierarchical and complex designs, identification of the appropriate level for tests and full reporting of outcomes
☐	☒	Estimates of effect sizes (e.g. Cohen's *d*, Pearson's *r*), indicating how they were calculated

Our web collection on statistics for biologists contains articles on many of the points above.

Software and code

Policy information about availability of computer code

Data collection	NA
Data analysis	Github web-link to manuscript code: https://github.com/shailajaAkella/Deciphering-neuronal-variability<br><br>Github repositories:<br>AllenInstitute/AllenSDK,<br>lindermanlab/ssm,<br>pillowlab,<br>luisitobarcito,<br>shailajaAkella/Directed-Information<br><br>Python Libraries:<br>NumPy v1.20.3 (van der Walt et al., 2011)<br>SciPy v1.7.3 (SciPy 1.0 Contributors et al., 2020)<br>Pandas v1.2.3 (McKinney, 2010)<br>scikit-learn v1.0.2 (Pedregosa et al., 2012)<br>SLEAP (Pereira et al., 2022)<br>statsmodels v0.13.0 (Seabold and Perktold, 2010)<br>Einops v0.7.0 (Rogozhnikov, 2022)<br>Jupyter v1.0.0. - https://jupyter.org/<br>Kneed v0.8.5 (Vela, P., & Ranieri, L., 2019)

Matplotlib v3.4.2 (Hunter, 2007)
Seaborn v0.11.2 (Waskom, 2021)
OpenCV v4.5.5 (Itseez, 2015)
UMAP v0.4.1 (McInnes, 2018)
Rastermap v0.1.3 (C. Stringer et al., 2024)

For manuscripts utilizing custom algorithms or software that are central to the research but not yet described in published literature, software must be made available to editors and reviewers. We strongly encourage code deposition in a community repository (e.g. GitHub). See the Nature Portfolio guidelines for submitting code & software for further information.

Data

Policy information about availability of data

All manuscripts must include a data availability statement. This statement should provide the following information, where applicable:

- Accession codes, unique identifiers, or web links for publicly available datasets
- A description of any restrictions on data availability
- For clinical datasets or third party data, please ensure that the statement adheres to our policy

The dataset analyzed in the paper is publicly available at: https://portal.brain-map.org/circuits-behavior/visual-coding-neuropixels

Research involving human participants, their data, or biological material

Policy information about studies with human participants or human data. See also policy information about sex, gender (identity/presentation), and sexual orientation and race, ethnicity and racism.

Reporting on sex and gender	NA
Reporting on race, ethnicity, or other socially relevant groupings	NA
Population characteristics	NA
Recruitment	NA
Ethics oversight	NA

Note that full information on the approval of the study protocol must also be provided in the manuscript.

Field-specific reporting

Please select the one below that is the best fit for your research. If you are not sure, read the appropriate sections before making your selection.

☒ Life sciences ☐ Behavioural & social sciences ☐ Ecological, evolutionary & environmental sciences

For a reference copy of the document with all sections, see nature.com/documents/nr-reporting-summary-flat.pdf

Life sciences study design

All studies must disclose on these points even when the disclosure is negative.

Sample size	Our study analyzes all the data collected under the "functional connectivity" stimulus set in the Visual Coding Neuropixels dataset. This set was chosen to study neuronal responses to the natural movie stimulus included within it.
Data exclusions	All the quality control metrics recommended by the authors of the public repository were applied and additional data inclusion criteria are described in the text.
Replication	All code and data required to reproduce the results of this study are provided in the manuscript.
Randomization	NA. This study utilized publicly available data.
Blinding	This study utilized publicly available data, making blinding during data collection and group allocation inapplicable.

Reporting for specific materials, systems and methods

We require information from authors about some types of materials, experimental systems and methods used in many studies. Here, indicate whether each material, system or method listed is relevant to your study. If you are not sure if a list item applies to your research, read the appropriate section before selecting a response.

Materials & experimental systems

n/a	Involved in the study
☒	☐ Antibodies
☒	☐ Eukaryotic cell lines
☒	☐ Palaeontology and archaeology
☐	☒ Animals and other organisms
☒	☐ Clinical data
☒	☐ Dual use research of concern
☒	☐ Plants

Methods

n/a	Involved in the study
☒	☐ ChIP-seq
☒	☐ Flow cytometry
☒	☐ MRI-based neuroimaging

Animals and other research organisms

Policy information about studies involving animals; ARRIVE guidelines recommended for reporting animal research, and Sex and Gender in Research

Laboratory animals	Mus musculus, strain, age and species are reported here: https://portal.brain-map.org/circuits-behavior/visual-coding-neuropixels
Wild animals	NA
Reporting on sex	NA
Field-collected samples	NA
Ethics oversight	Mice were maintained in the Allen Institute for Brain Science animal facility and used in accordance with protocols approved by the Allen Institute's Institutional Animal Care and Use Committee.

Note that full information on the approval of the study protocol must also be provided in the manuscript.

Plants

Seed stocks	*Report on the source of all seed stocks or other plant material used. If applicable, state the seed stock centre and catalogue number. If plant specimens were collected from the field, describe the collection location, date and sampling procedures.*
Novel plant genotypes	*Describe the methods by which all novel plant genotypes were produced. This includes those generated by transgenic approaches, gene editing, chemical/radiation-based mutagenesis and hybridization. For transgenic lines, describe the transformation method, the number of independent lines analyzed and the generation upon which experiments were performed. For gene-edited lines, describe the editor used, the endogenous sequence targeted for editing, the targeting guide RNA sequence (if applicable) and how the editor was applied.*
Authentication	*Describe any authentication procedures for each seed stock used or novel genotype generated. Describe any experiments used to assess the effect of a mutation and, where applicable, how potential secondary effects (e.g. second site T-DNA insertions, mosiacism, off-target gene editing) were examined.*
